# Controlled mechanical ventilation tactics in patients with polytrauma during interhospital transportation to the specialized center

**DOI:** 10.1186/cc11067

**Published:** 2012-03-20

**Authors:** A Shatalin, S Kravtsov, V Agadzhanyan, D Skopintsev

**Affiliations:** 1 Federal State Budgetary Medical Prohylactic Institution, Leninsk-Kuznetsky, Russia; 2 Federal State Budgetary Medical Prohylactic Institution 'Scientific Clinical Center of the Miners Health Protection', Leninsk-Kuznetsky, Russia

## Introduction

This study is an analysis of the influence of controlled mechanical ventilation (CMV) with PEEP in conditions of pneumocompression of the Chestnut antishock suit on the hemodynamics and blood oxygenation in patients with polytrauma during interhospital transportation.

## Methods

Seventy-two patients with polytrauma complicated by II and III stage ARDS were included in the study. The mean age was 33 ± 2 years. All patients were divided into two equal groups. The control group (CG) CMV was carried out with no PEEP. The experimental group (EG) CMV was carried out with PEEP 8 to 10 mbar. Both groups received the CMV regimen with V_t _7 ml/kg, P_max _30 mbar. The injury severity according to the ISS was 37.6 ± 1 points in the EG and 39.1 ± 1 in the CG. The transportation time was 135 ± 7 minutes, the distance was 136 ± 10 km. Immobilization in the lower extremity fractures and pelvis fractures was carried out using the Chestnut suit with pneumocompression over the damaged parts of the body of 40 mmHg and over the remaining parts of the body of 20 mmHg. Statistical analysis was performed using Statistica 6.1. We used Student's criterion.

## Results

In the EG there were the high values of SpO_2 _during all observation periods and PaO_2_/FiO_2 _after completion of the transportation in 1 and 12 hours (*P *< 0.05). PaCO_2 _in the EG was lower after completion of the transportation in 1 and 12 hours compared to the CG (*P *< 0.05). In the EG the value of FiO_2 _decreased from 0.5 ± 0.01 in the early examination to 0.4 ± 0.01 in 12 hours after transportation. In the CG, FiO_2 _did not change. Hemodynamics differences between the groups were not documented, except for HR (*P *> 0.05). Tachycardia was less expressed in the EG. The difference from the CG according to this index occurred 12 hours after completion of the transportation, 83 ± 1 and 87 ± 0.7 beats/minute respectively (*P *< 0.05). The lactate rate was lower in the EG during all periods of observation (*P *< 0.05). After completion of the transportation, the lactate rate in the EG was 2.2 ± 0.1 mol/l and in the CG was 2.7 ± 0.1 mol/l.

## Conclusion

Use of CMV with PEEP in patients with polytraumacomplicated ARDS provided more expressed improvement of the blood oxygenation. Improvement of the blood gas exchange was accompanied by lactate decrease in both groups: by 24% in the EG, and by 13% in the CG. Application of the Chestnut allowed one to level the hemodynamic disorders using CMV with PEEP by means of preload maintenance and of the systolic output as a consequence.

